# Exploring Ortho–Para Hydrogen Conversion Catalysts Based on Surface Electric Field Gradient

**DOI:** 10.1021/acs.jpclett.6c00357

**Published:** 2026-03-12

**Authors:** Hiroshi Mizoguchi, Yuichi Shirako, Shusaku Shoji, Hideki Abe, Takeshi Fujita, Hideo Hosono

**Affiliations:** † Research Center for Materials Nanoarchitectonics (MANA), 52747 National Institute for Materials Science (NIMS), Tsukuba, Ibaraki 305-0044, Japan; ‡ Center for Green Research on Energy and Environmental Materials, National Institute for Materials Science (NIMS), Tsukuba, Ibaraki 305-0044, Japan; § Kochi University of Technology, 185 Miyanokuchi, Tosayamada, Kami, Kochi 782−8502, Japan; ∥ MDX Research Center for Element Strategy, International Research Frontiers Initiative, Institute of Science Tokyo, 4259 Nagatsuta, Midori-ku, Yokohama 226-8503, Japan

## Abstract

According to our hypothesis that ortho (O) to para (P) hydrogen conversion is promoted by an inhomogeneous electric field on the surface of the insulating oxide with high ionicity, we searched for OP conversion catalysts using lattice energy as an indicator of high ionicity. As a result, we found new oxide catalysts, including SiO_2_, γ-Al_2_O_3_, and CeO_2_ combined with 3d late transition metal cocatalysts. The fraction of para-H_2_ on 5%Fe-loaded SiO_2_ powder reached 50% (equilibrium value at 77 K) within 20 min. The activities of these catalysts are significantly superior to those of benchmark catalysts, such as Mn_3_O_4_. The cocatalyst nanoparticles of 1–12 nm size dispersed on the oxide catalysts adsorb hydrogen well without dissociating it. The nuclear spin state of ortho-H_2_ adsorbed at the asymmetric site (nonzero electric field gradient) of the oxide surface is thermally excited by nuclear quadrupole interaction, and the OP conversion rate is increased by nuclear spin relaxation.

## Graphical abstract

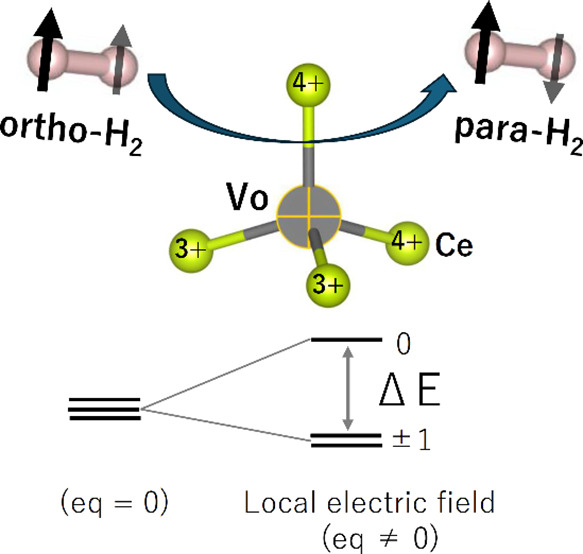

Recently, the demand for liquid hydrogen as an energy carrier for transportation and storage in hydrogen economy has increased owing to its potential for high volumetric and energy storage densities.^1^ However, there is a difficulty inherent to hydrogen. The homonuclear diatomic molecule H_2_ possessing ^1^H nuclei (I = 1/2), has two nuclear spin isomers of ortho (O; J = 1) and para (P; J = 0). Normal H_2_ is a mixture of these nuclear spin isomers with an O/P ratio of 3 at room temperature. The equilibrium O/P ratio follows the Maxwell–Boltzmann distribution and changes significantly with temperature (Figure S1 in the Supporting Information), whereas p-H_2_ occupies the rotational ground state of J = 0 and is more stable than o-H_2_ (J = 1) by an energy difference of 2B (B: rotational constant). However, the ortho to para (OP) conversion does not proceed without the help of a catalyst, despite the downhill reaction.^2^ Liquid hydrogen obtained from the liquefaction process contains a high proportion of o-H_2_, which causes boil-off, leading to the loss of liquid hydrogen. Hydrogen is the lightest element, resulting in a high rotational energy (2B = 15 meV), which is higher than the vaporization energy (ΔH_vap_ = 9.4 meV). To overcome this obstacle, catalysts that promote OP conversion before liquefaction are required.

Many catalysts for OP conversion, including Fe_2_O_3_·*n*H_2_O, γ-Fe_2_O_3_, and Cr_2_O_3_, have been proposed so far.^3–11^ Although the exact origin for the conversion has not yet been elucidated, there are two representative models depending on the type of surface. One is an inhomogeneous magnetic field appearing on the surfaces of antiferromagnetic materials containing magnetic ions such as Fe or Cr ions, which generate the magnetic dipole–dipole or Fermi contact interaction.^12,13^ The other is an electric field appearing on the surfaces of ionic compounds having no magnetic ions. (Stark effect)^14^ Recently, we have proposed a working hypothesis, determined through the search of the catalysts.^15^ A key discovery is that whereas metallic materials are inactive, active catalysts are in most cases insulators with the ionic bonding characteristic, whose cations have an ionic radius smaller than the interatomic distance (0.74 Å) of the H_2_ molecule. Highly charged cations with small radii on insulating surfaces can generate an electrostatic field extending over physisorbed hydrogen, with a gradient shorter than the internuclear distance, causing hydrogen to behave as a molecule with two distinct nuclei. Here in this letter, we report on a new high-activity OP conversion catalyst explored on the basis of this working hypothesis.

According to our hypothesis, OP conversion is promoted by an inhomogeneous electric field on the surface of the insulating oxide with a high ionicity composed of small ions with a large valence. Considering the large negative charge of anions, oxides are promising candidates as catalysts. Hydrogen has amphoteric character, and alters its valence state from positive (cationic) to negative (anionic) through electron transfer, depending on its chemical environment, owing to its electronegativity.^16^ The H_2_ molecule dissociates heterolytically at room temperature or above on the surfaces of insulating oxides where the distribution of anions/cations is similar to a checkerboard pattern,^17,18^ and we can expect various modulations on the surfaces of oxides, caused by the inhomogeneous electric field. Thus, we expect that lattice energy will serve as an indicator for the search for catalysts because it is one of the main factors for stabilizing ionic crystals. Table S1 summarizes the calculated lattice energy for representative oxides. The primary factors governing lattice energy are the charge state, the distance between charges, and the degree of ion packing. Spinel-type oxides have the tendency to have high lattice energy values. In fact, oxides exhibiting high catalytic activity, such as Mn_3_O_4_ and γ-Fe_2_O_3_ (= Fe_2.67_O_4_) are of the spinel type, suggesting that the lattice energy is a useful indicator for catalyst development. Therefore, we selected SiO_2_, Al_2_O_3_, and CeO_2_ as the candidates on the basis of their high lattice energy values. While the Si^4+^ ion is smaller with higher valence, α-SiO_2_ (quartz type) does not have a very high lattice energy because of the loose packing derived from the two coordination of the O^2–^ ion. As for Al_2_O_3_, we selected the type with γ-polymorphism, having a lower density (3.64 gcm^–3^) than that with α-polymorphism (4.00 gcm^–3^). We describe the crystal structures of these candidates. In amorphous SiO_2_, SiO_4_ tetrahedra connect to each other through corner sharing to form an amorphous structure with a lower packing feature. γ-Al_2_O_3_ (= Al_2.67_O_4_) adopts a defect spinel-type structure with plenty of crystallographic voids.^19^ Figure 1a shows the *B*_2_O_4_ sublattice in a normal spinel-type crystal structure with the *AB*_2_O_4_ composition, where *A* and *B* cations occupy the tetrahedral and octahedral sites, respectively. This structure consists of alternating layers of a closed-packed layer of O ions, stacked along the [111] direction in an ABCABC sequence, and Al1, Al2, or Al3 ions occupy the crystallographic cavity site between the layers with an occupancy smaller than 1, resulting in cationic deficiency. Whereas the lattice energy of the γ-phase must be slightly smaller than that of the α-phase, the cationic vacancy and low atomic density of the γ-phase are expected to enhance catalytic activity, because of the increase of active center. Figure 1b shows the crystal structure of CeO_2_. Ce^4+^ ion coordinates with eight O^2–^ ions, and the O^2–^ ion in the tetrahedral symmetry (*T*_d_) site coordinates with four Ce^4+^ ions, as shown in Figure 2a.

**Figure 1:**
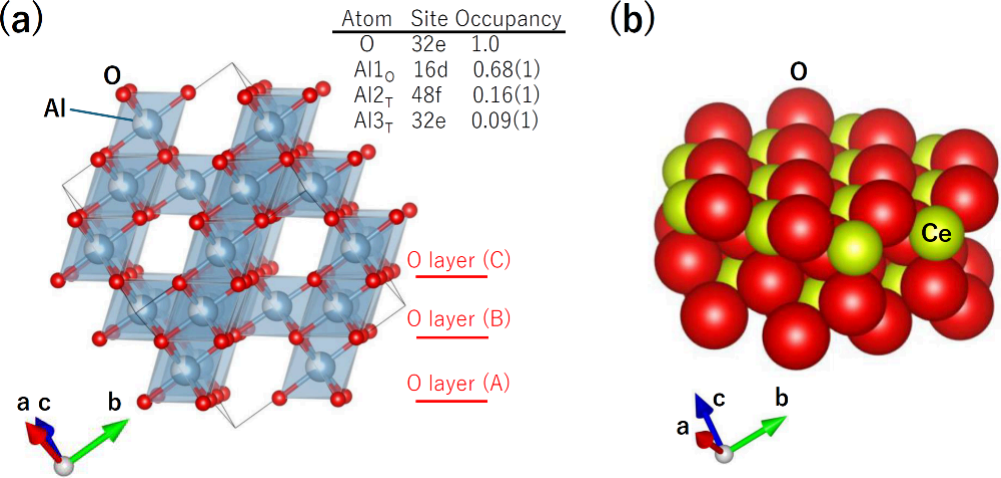
(a) (Al1_O_)_1.36_O_4_ sublattice in γ-Al_2_O_3_ (= Al_2.68_O_4_ = (Al2_T_, Al3_T_)_1.32_(Al1_O_)_1.36_O_4_).^19^ Al_O_ and Al_T_ are the Al ions in the octahedral and tetrahedral coordination sites, respectively. In the framework, AlO_6_ octahedra share edges and corners to form a three-dimensional network. (b) Crystal structure of CeO_2_.

**Figure 2:**
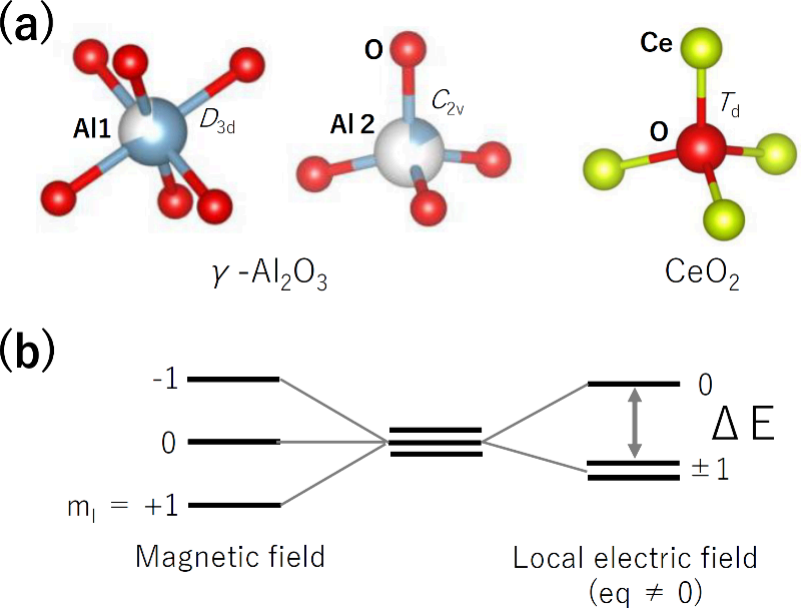
(a) Local coordination of defective sites in γ-Al_2_O_3_ and CeO_2_. (b) Energy splitting ΔE of nuclear spin levels for a nucleus of I = 1 (q: electric field gradient at the nuclear position).

Figure S3a shows the Raman spectra for CeO_2_ loaded with 5 mol %Ni, as an example. The sharp peaks at 354.4 and 588.4 cm^–1^ are ascribed to J = 0 (p-H_2_) and J = 1 (o-H_2_), respectively, and the fraction of p-H_2_ evaluated from the intensity ratio is 25% before exposure to the catalyst even at 77 K. As soon as H_2_ gas is exposed to the catalyst, the intensity ratio of the peaks begins to change, reaching 50% (equilibrium value at 77 K). Figure S3b shows the time course of OP conversion on these oxides at 77 K. The data on Mn_3_O_4_ or Fe_2_O_3_ is also shown as a reference^15^ in Figure S3c. We calculated the reaction rate constant (k) from the time course data. As an example, Mn_3_O_4_ was estimated to have k = 10.5(2.3) h^–1^, and an equilibrium fraction value (50%) was achieved after ∼30 min. Figure S3 shows that the catalytic activities of SiO_2_, Al_2_O_3_, and CeO_2_ are significantly inferior to that of Mn_3_O_4_. We considered that the promotion of low-temperature adsorption of H_2_ on the catalyst surface that does not involve the H_2_ dissociation process is key for OP conversion. In general, hydrogen adsorption on insulating oxide surfaces is more difficult than the adsorption of metallic compounds. The observed low activities of SiO_2_, Al_2_O_3_ and CeO_2_ appear to originate from the difficulty in hydrogen adsorption. To overcome this difficulty, we loaded a small amount (5 mol %) of a 3d late transition metal (TM) on supported oxide catalysts by the impregnation method. It is noted that these 3d TMs are generally inactive for OP conversion,^15^ whereas they cause H_2_ dissociation on the surface. Figure 3a shows the time course of the catalytic reaction of these samples, and the obtained rate is summarized in Table 1, together with the BET surface area. It is obvious that the addition of TM as a cocatalyst significantly improved the catalytic activity: 21.3 (0.8) h^–1^ for SiO_2_:Fe, 29.4 (7.5) h^–1^ for Al_2_O_3_:Co, 15.2 (2.5) h^–1^ for CeO_2_:Fe, and 6.2 (0.9) h^–1^ for CeO_2_:Ni. As an example, the fraction of p-H_2_ on SiO_2_/Fe powder reached 50% (equilibrium value at 77 K) within 20 min. The catalytic activity of TM-supported oxides containing environmentally benign elements was superior to those of reference oxides, including Mn_3_O_4_, as shown in Figure 3b. The order of the obtained activities (SiO_2_ ≈ Al_2_O_3_ > CeO_2_) matched moderately those of the lattice energies.

**Table 1:** Properties of SiO_2_, Al_2_O_3_, and CeO_2_-Based Catalysts

Catalyst	Reaction rate at 77 K (h^–1^)	Surface area (m^2^ g^–1^)
SiO_2_	-	27.4
5 mol % Fe	21.3(0.8)	-
γ-AlO_1.5_	-	169
5 mol % Co	29.4(7.5)	-
5 mol % Ni	9.1(1.3)	-
CeO_2_	-	138.1
5 mol % Fe	15.2(2.5)	-
5 mol % Ni	6.2(0.9)	117.6
Mn_3_O_4_	10.5(2.3)	18.9
Fe_2_O_3_	1.3(0.1)	8.7

**Figure 3:**
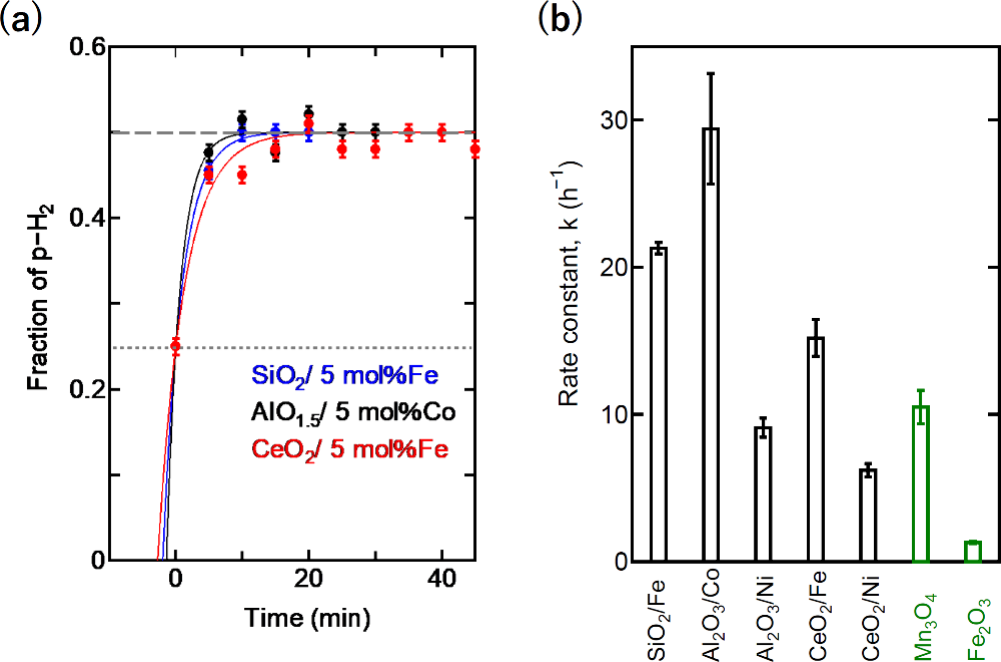
(a) Trends of the OP conversion at 77 K by SiO_2,_ γ-Al_2_O_3_, and CeO_2_-based catalysts. The dotted and dashed lines indicate the equilibrium values at 300 and 77 K, respectively. (b) Rate constants of OP conversion by representative catalyst materials.

We characterized the active catalysts in order to clarify the effect of the TM cocatalyst. Figure S4 shows the powder XRD patterns of the catalysts. Although we observe the diffraction pattern originating from γ-Al_2_O_3_ and CeO_2_, there was not much information about the diffraction by TM cocatalysts because of their small loaded amounts. We also observed the microstructure by TEM. Figure 4(a) shows an STEM image of the SiO_2_/Fe catalyst, with FeO_*x*_ particles confirmed by EDS mapping in Figure 4(b). Figure 4(c) shows CoO_*x*_ nanoparticles well dispersed in the Al_2_O_3_/Co catalyst. The electron diffraction of Al_2_O_3_/Co indicates the coexistence of Co, CoO, and Co_3_O_4_ (not shown). Figure 4(e) shows a TEM image of the CeO_2_/Ni catalyst, with metallic Ni particles confirmed by EDS mapping in Figure 4(d). Figure S5 shows the size distribution of TM species for selected catalysts. The sizes of these particles are 1–5, 3–12, and 2–5 nm for Fe, Co, and Ni species, respectively, suggesting that these species show superparamagnetism, judging from the size. The oxidation states estimated from STEM–EDS results were consistent with those estimated from the chemical shift in XPS spectra, as shown in Figure S6. The valence state of the TM cocatalyst decreased from Fe to Ni in the periodic table, which corresponds to the tendency of the workfunction of TMs.^20^ No influence of the basicity of the oxide (supporters) has been observed. In the case of CeO_2_/Ni, the reduction of Ce ion was confirmed in Ce 3d XPS, as shown in Figure S6­(c). In fact, the color of CeO_2_-based catalysts was changed from cream yellow to dark brown by low-temperature heat treatment under an Ar–5%H_2_ atmosphere, suggesting the formation of Ce^3+^ ions. Figure S7 shows the H_2_-TPD profiles of the oxides with and without the cocatalyst. Obtained information obtained from the curves is summarized in Table S2. For SiO_2_/Fe, significant H_2_ desorption was observed at temperatures above 300 °C, from which the composition was determined to be SiO_2_/Fe_0.05_/(H_2_)_0.0034_. This hydrogen content represents an increase of more than 15 times compared with the sample without the cocatalyst [SiO_2_(H_2_)_0.0002_], indicating that the Fe cocatalyst markedly improves hydrogen adsorption.

**Figure 4:**
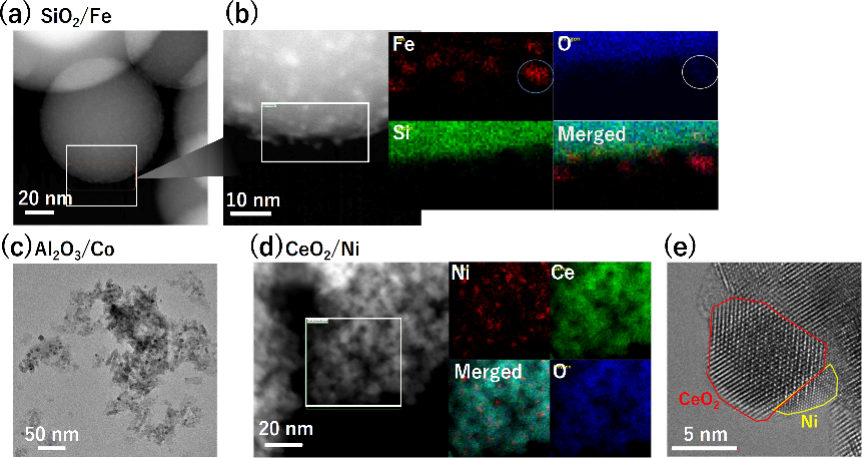
Scanning TEM (STEM) and energy-dispersive X-ray spectroscopy (EDS) mapping of representative catalysts. (a) Low-magnification STEM image of SiO_2_/Fe, showing large oxidic particles. (b) High-magnification STEM image and EDS elemental maps (Fe, O, and Si), and merged map, confirming the oxide nature of Fe species dispersed on SiO_2_. (c) TEM image of Al_2_O_3_/Co, showing particles of CoO_*x*_ species. (d) High-magnification STEM image of CeO_2_/Ni, EDS elemental maps in square region (Ni, Ce, and O), and merged map, showing well dispersed metallic Ni particles. (e) TEM lattice image showing CeO_2_ nanocrystal with a diameter of ∼10 nm, which is in close contact with a metallic Ni particle.

The OP conversion does not proceed without a catalyst, despite the downhill reaction with an energy difference of 2B = 15 meV. We discuss the main factor that promotes OP conversion. Since it involves the conversion between nuclear spin isomers, directly stimulating the nuclear spin of the ^1^H atom must be effective. This requires modulation of the nuclear spin levels using a magnetic or electric field. In 1953, Reif and Purcell reported the nuclear magnetic resonance of o-H_2_ dispersed in solid hydrogen in zero magnetic fields.^21^ They observed the absorption of radio waves with ΔE = 6.8 × 10^–10^ eV. This reminds us of its similarity to the nuclear quadrupole resonance in zero magnetic fields and the Mossbauer effect, which are applicable to nuclei with I ≥ 1. As a trial, we regard o-H_2_ (J = 1) as a single nucleus (I = 1). A single nucleus with I = 1 exhibits an ellipse-shaped charge distribution, giving rise to an electric quadrupole, which induces the splitting of nuclear spin levels, depending on the chemical environment [electric field gradient (EFG): eq] (Figure 2b). The energy splitting (ΔE) is proportional to this eq and increases in low-symmetry environments including surfaces. The thermal energy at 77 or 25 K is sufficient for excitation of nuclear spin levels. Therefore, it is possible to excite nuclear spins directly, only when o-H_2_ locates in low-symmetry environments. Thus, we may expect the relaxation from J = 1 (o-H_2_) to J = 0 (p-H_2_), i.e., enhancement of OP conversion. Here, we need to examine the details of the crystal structure of our oxides because the energy splitting depends highly on the local environments around the adsorbed o-H_2_. As an example, the O-site in CeO_2_ with the fluorite-type crystal structure has *T*_d_ symmetry with eq = 0 (that is, ΔE = 0), as shown in Figure 2a. The equilibrium oxygen partial pressure for the Ce_2_O_3_/CeO_2_ oxidation reaction is ∼1 × 10^–90^ atm at 573 K, according to the Ellingham diagram,^22^ which is impossible to realize under our conventional experimental condition. It is difficult to realize the O deficiency in CeO_2_. However, our reagent consists of nanoparticles with a diameter of ∼10 nm. The formation of Ce^3+^ ions on the surface is expected because of the large contribution of the surface energy of nanoparticles, which has been confirmed by TEM observation.^23^ CeO_2_ is a band insulator with a bandgap of ∼4 eV. The conduction band minimum (CBM) originates primarily not from Ce 5d but from 4f states, whereas the Ce^4+^ ion has the (5d4f)^0^ electronic configuration. Two electrons generated by an O vacancy are trapped on the 4f levels to form two Ce^3+^ ions, without forming free carriers at the CBM. The symmetry of the O vacancy site surrounded by two Ce^4+^ and two Ce^3+^ ions decreases from the *T*_d_ symmetry and the EFG exhibits its maximum value at n = 2 in a local environment surrounded by Ce^4+^_4–*n*_Ce^3+^_*n*_ (*n* = 0, 1, 2, 3, or 4), according to a point charge model.^24^ The O vacancy with a diameter of 2.42 Å is expected to accommodate a hydrogen molecule. It is expected that a large number of such low-symmetry sites will exist on the surface and near-surface regions of CeO_2_ nanocrystals. Similarly, γ-Al_2_O_3_ (= Al_2.67_O_4_) has a lot of cationic deficiency sites, as expected from its chemical composition and low density (Figure 2a), which possibly form an inhomogeneous electric field on the surface. We found noble OP conversion catalysts showing highly catalytic activities at 77 K by searching based on our working hypothesis related to ionic bonding characteristics in insulating oxides. Next, the design and control of surface defects, considering the crystallographic symmetry in insulating oxides, will be our next focus.

## Supplementary Material



## Notes

Lattice energy, temperature dependence of hydrogen isomers, catalyst evaluation setup, time course of catalytic activity, powder XRD, size distribution histograms, XPS, and H_2_-TPD (PDF)
