# Supplementary material for: Exploring Ortho–Para Hydrogen Conversion Catalysts Based on Surface Electric Field Gradient
Source: J Phys Chem Lett. 2026 Mar 12;17(12):3701–5. doi: 10.1021/acs.jpclett.6c00357 (PMC13034456; doi:10.1021/acs.jpclett.6c00357)
Supplement: Supplementary file 1 [file jz6c00357_si_001.pdf]

Supporting Information

Exploring ortho–para hydrogen conversion catalysts based on surface electric field gradient

Hiroshi Mizoguchi,[1] Yuichi Shirako,[2] Shusaku Shoji,[2] Hideki Abe,[2]

Takeshi Fujita,[3] Hideo Hosono[1, 4]

[1]Research Center for Materials Nanoarchitectonics (MANA), National Institute for Materials Science (NIMS), Tsukuba, Ibaraki 305-0044, Japan

[2]Center for Green Research on Energy and Environmental Materials, National Institute for Materials Science (NIMS), Tsukuba, Ibaraki 305-0044, Japan

[3]Kochi University of Technology, 185 Miyanokuchi, Tosayamada, Kami, Kochi 782–8502, Japan

[4]MDX Research Center for Element Strategy, International Research Frontiers Initiative, Institute of Science Tokyo, 4259 Nagatsuta, Midori-ku, Yokohama 226-8503, Japan

Corresponding author footnote: Hideo Hosono, hosono@mces.titech.ac.jp

Hideki Abe, ABE.Hideki@nims.go.jp

Hiroshi Mizoguchi, MIZOGUCHI.Hiroshi@nims.go.jp

(i) Synthesis

A series of metal-supported oxide catalysts were prepared using SiO_2, Al_2O_3, and CeO_2 as supports, and Fe, Co, and Ni as metal species. The reactants were SiO_2 (DENKA, UFP-30), γ-Al_2O_3 (Nikki, JRC-ALO-6), CeO_2 (99.9%, Daiichikigenso, type-A), Mn_3O_4 (99.9%, Kishida Chemical Co.), Fe_2O_3 (99.9%, Aldrich), $Fe(acac)_3$ (98.0%, Tokyo Chemical Industry), $Co(NO_3)_2 \cdot 6H_2O$ (99.9%, Kojundo Chemical Laboratory), and $Ni(NO_3)_2 \cdot 6H_2O$ (99.9%, Fujifilm Wako). For each sample, the metal loading amount was adjusted to 5 mol% with respect to the total moles of the support. The catalysts were synthesized by an impregnation method, in which the oxide support was suspended in an aqueous solution of the corresponding metal salt. In the case of Fe cocatalyst, $Fe(acac)_3$ was mixed using a agate mortar, together with oxide catalyst. After impregnation, the materials were dried thoroughly and thermally reduced in a furnace under Ar–5% H_2 gas. The reduction was carried out at 300 °C for 3 h.

(ii) Characterization

The resulting catalysts were characterized by various techniques. Crystalline phases were analyzed by powder X-ray diffraction (XRD, Miniflex II, RIGAKU, Japan). The lattice energy was calculated using MADEL.[1] The Brunauer–Emmett–Teller (BET) surface areas were obtained from the BET plot at $P/P_0 < 0.3$. Transmission electron microscopy (TEM) imaging was performed using a JEM-ARM200F NEOARM microscope (JEOL) equipped with aberration correctors for image and probe-forming lens systems (CEOS GmbH). Energy-dispersive X-ray spectroscopy analysis was conducted using a JED-2300T (JEOL) detector. TEM and scanning TEM (STEM) observations were performed at an accelerating voltage of 200 kV. X-ray photoelectron spectroscopy (XPS) measurements were performed with a Quantes (ULVAC-PHY Inc., Japan) with a monochromatic Al K_a X-ray source (hν = 1486.6 eV) operated at 15 kV. The temperature-programmed desorption of H_2 (H_2-TPD) was measured

using a TPD-1-Atw (Microtrac BEL). 0.2 g of a sample was placed in a quartz tube and treated at 300 °C for 1 h under a He flow rate of 50 mL/min. The temperature was then lowered to 50 °C while maintaining the He atmosphere, and He–5%H_2 was introduced at 100 mL/min for 0.5 h to allow its adsorption onto the sample. The sample was then heated to 700 °C, and the desorption of H_2 was detected using a Q-MS.

(iii) Catalyst evaluation

The ortho-to-para hydrogen conversion activity of the catalysts was examined at 77 K using a custom-built batch-type reactor system, as shown in **Figure S2**. The setup consisted of a plunger pump for continuous H_2 circulation and a gas-tight Raman cell for in situ spectral monitoring. Commercially available H_2 gas (99.99%) was used. Approximately 300 mg of a catalyst was placed in a borosilicate glass tube (6 mm inner diameter), and the tube was mounted onto the reactor, followed by evacuation to a base pressure of 10 Pa. Subsequently, H_2 gas was introduced until the system pressure reached 80 kPa. The gas was circulated throughout the catalyst bed and Raman cell in series, enabling the real-time observation of the OP ratio using a Raman spectrometer (JASCO RMP-510).

Reference

1. Momma, K.; Izumi, F., VESTA 3 for three-dimensional visualization of crystal, volumetric and morphology data. *J. Appl. Crystallogr.* **2011**, *44* (6), 1272-1276.
2. Fujimori, A., Correlation effects in the electronic structure and photoemission spectra of mixed-valence cerium compounds. *Phys. Rev. B* **1983**, *28* (8), 4489-4499.

Table S1. Calculated lattice energies of representative oxides. In the case of γ-Al_2O_3, there are several sites having cationic deficiency, as shown in **Fig. 1a**. This prevents the lattice energy calculation. In the place of γ phase, we show that of α-polymorphism.

Crystal structure	Oxide	Lattice energy (eV)
Corundum	α-Al_2O_3	188.9
	Fe_2O_3	178.4
Quartz	α-SiO_2	157.5
Fluorite	CeO_2	123.6
Rocksalt	MgO	47.7
Wurtzite	ZnO	47.8
Spinel	$MgAl_2O_4$	236.2
	Mn_3O_4	225.4

Table S2. Desorption temperature and uptake of H_2-TPD profiles (m/z = 2) of SiO_2-, Al_2O_3-, and CeO_2-based catalysts, shown in **Fig. S7**.

Catalyst	Desorption temperature (℃)<br>Uptake（μmol/g）
SiO_2	452<br>3.4
5mol%Fe	469 (700)<br>38 18
γ-$AlO_{1.5}$	257 375 487 538<br>0.5 6.9 2.3 5.2
5mol%Co	260 389 506<br>0.7 6.8 5.2
CeO_2	418<br>6.2
5mol%Ni	268 391<br>1.0 2.7

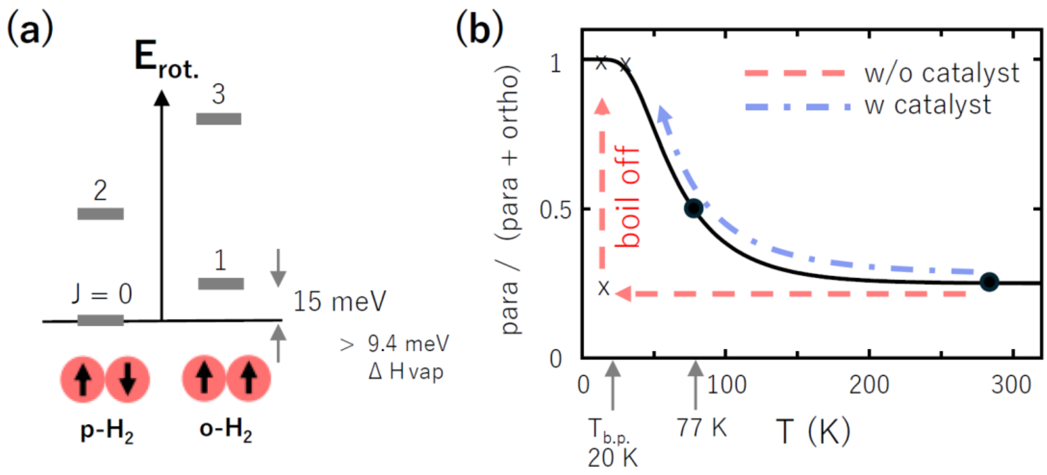

Fig. S1. (a) Rotational energy levels of molecular hydrogen. (b) Temperature dependence of equilibrium concentration of p-H_2.

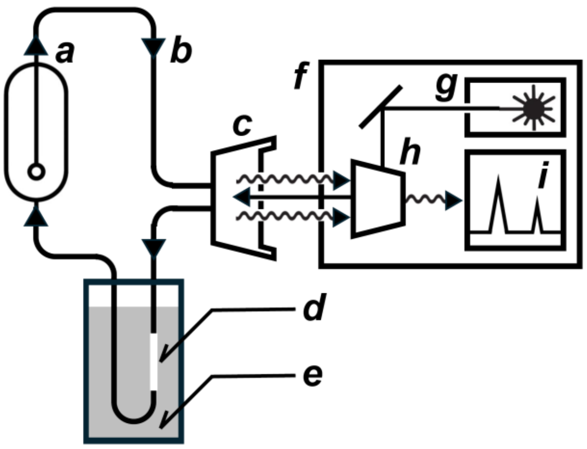

Fig. S2 Schematic of catalytic converter for nuclear spin flipping in hydrogen gas. a: plunger pump; b: hydrogen gas stream; c: gas cell; d: catalyst sample; e: liquid nitrogen; f: Raman spectrometer; g: laser source (532 nm, SHG of Nd: YAG); h: optical filter; i: spectrometer.

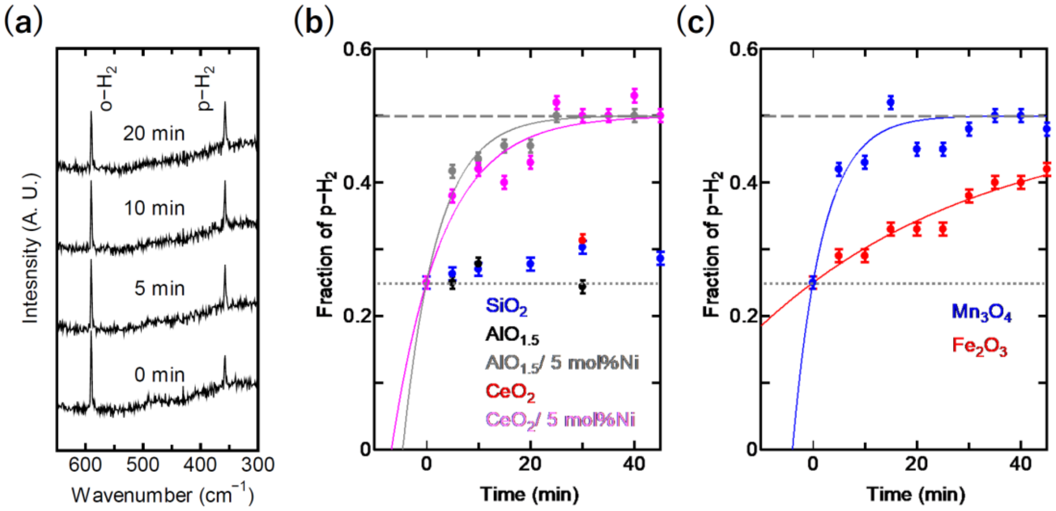

Fig. S3 (a) Time evolution of Raman spectra for the OP conversion over CeO_2/Ni catalyst at 77 K. Trends of the OP conversion at 77 K by (b) SiO_2, Al_2O_3, and CeO_2-based catalysts, and (c) reference catalysts. Exponential fitting was performed on experimentally obtained data to estimate the rate constant (k). The equation used was y = 0.50-0.25*exp(-k*x/60).

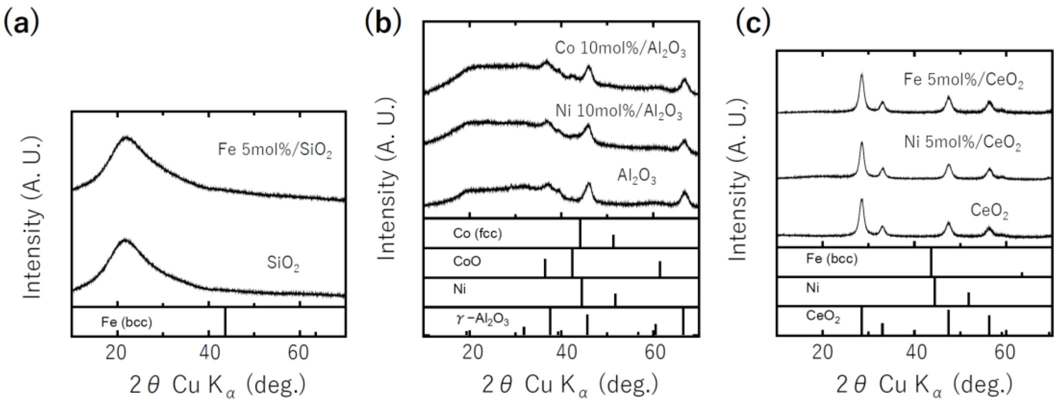

Fig. S4 Powder XRD patterns of (a) SiO_2, (b) Al_2O_3, and (c) CeO_2-based catalysts.

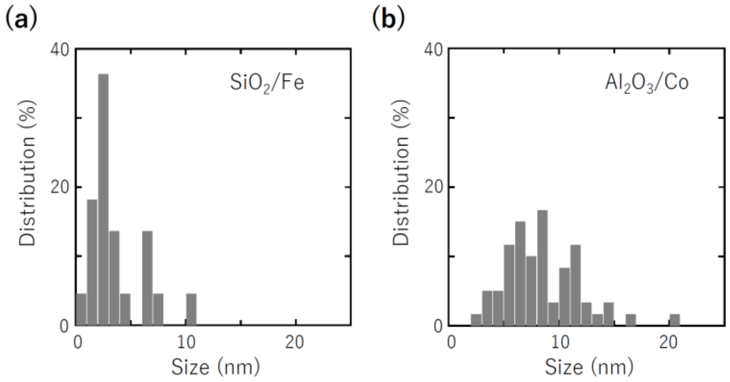

Fig. S5. Transition metal size distribution of (a)SiO_2/Fe and (b)Al_2O_3/Co catalysts.

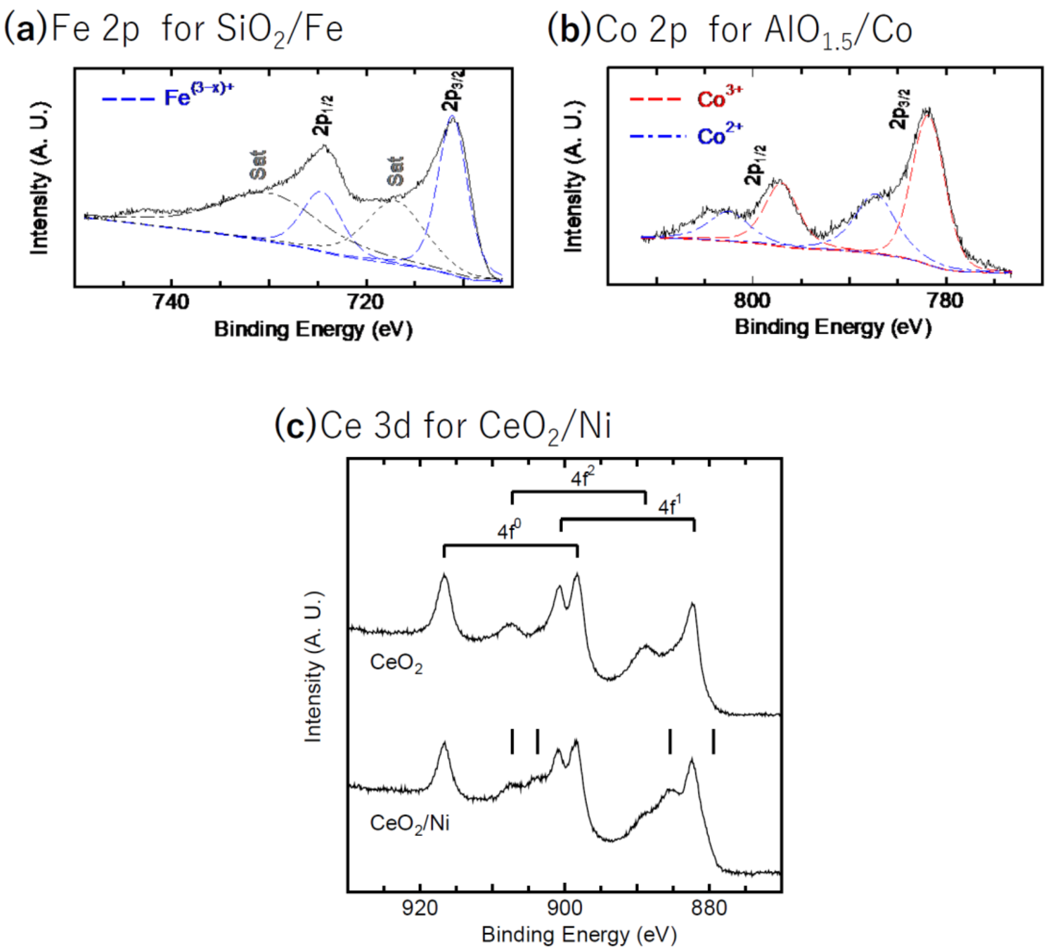

Fig. S6 XPS spectra of (a) SiO_2/Fe, (b) Al_2O_3/Co, and (c)CeO_2/Ni catalysts. These spectra indicate the weak oxidation of Fe and Co in the surface region. On the other hand, the Ce 3d XPS spectrum of the Ce^{4+} ion in (c) shows a complicated structure, owing to the final state effect[2]. Several weak peaks indicated by bars appeared for the catalyst with heat treatment under Ar–5%H_2. These peaks indicate the formation of Ce^{3+} by the reduction of CeO_2 nanocrystals.

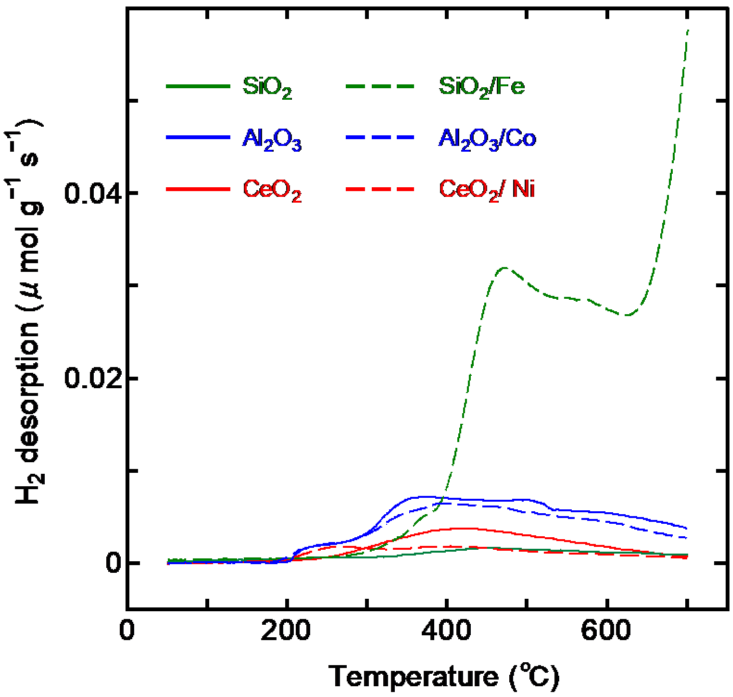

Fig. S7 H_2-TPD profiles (m/z = 2) of SiO_2, Al_2O_3, and CeO_2-based catalysts. 0.2 g of a sample was placed in a quartz tube and treated at 300 °C for 1 h under a He flow rate of 50 mL/min. The temperature was then lowered to 50 °C while maintaining the He atmosphere, and He–5%H_2 was introduced at 100 mL/min for 0.5 h to allow its adsorption onto the sample. The sample was then heated to 700 °C, and the desorption of H_2 was detected. The information obtained from this figure is summarized in **Table S2**.
